# Increased functional dynamics in civil aviation pilots: Evidence from a neuroimaging study

**DOI:** 10.1371/journal.pone.0234790

**Published:** 2020-06-18

**Authors:** Xi Chen, Quanchuan Wang, Cheng Luo, Yong Yang, Hao Jiang, Xiangmei Guo, Xipeng Chen, Jiazhong Yang, Kaijun Xu

**Affiliations:** 1 Institute of Flight Technology, Civil Aviation Flight University of China, Guanghan, China; 2 Key Laboratory for NeuroInformation of Ministry of Education, School of Life Science and Technology, University of Electronic Science and Technology of China, Chengdu, China; Tongii University, CHINA

## Abstract

Civil aviation is a distinctive career. Pilots need to monitor the entire system in real time. However, the psychophysiological mechanism of flying is largely unknown. The human brain is a large-scale interconnected organization, and many stable intrinsic large-scale brain networks have been identified. Among them are three core neurocognitive networks: default mode network (DMN), central executive network (CEN), and salience network (SN). These three networks play a critical role in human cognition. This study aims to examine the dynamic properties of the three large-scale brain networks in civil aviation pilots. We collected resting-state functional magnetic resonance imaging data from pilots. Independent component analysis, which is a data-driven approach, was combined with sliding window dynamic functional connectivity analysis to detect the dynamic properties of large-scale brain networks. Our results revealed that pilots exhibit an increased interaction of the CEN with the DMN and the SN along with a decreased interaction within the CEN. In addition, the temporal properties of functional dynamics (number of transitions) increased in pilots compared to healthy controls. In general, pilots exhibited increased between-network functional connectivity, decreased within-network functional connectivity, and a higher number of transitions. These findings suggest that pilots might have better functional dynamics and cognitive flexibility.

## Introduction

Civil aviation is a distinctive career. Pilots work in a complex, dynamic information environment. They must be aware of all the relevant information regarding this environment and recognize their meaning and importance. Moreover, they should understand what is going on and to go on [1]. This process refers to situational awareness [2]. Inadequate situation awareness might cause errors, even in aircraft accidents. These processes include perceptual, memory, planning, assumption, and expectation processes, which can be summarized as internally oriented mental processes, goal-directed processes, and ongoing evaluation [3, 4]. Their work involves virtually the entire brain. However, relatively few studies have focused on the neurophysiological mechanism of flying. Pilots work in a complex, dynamic information environment. They must be aware of all the relevant information regarding this environment and recognize their meaning and importance. Moreover, they should understand what is going on and to go on [1]. This process refers to situational awareness [2]. Inadequate situation awareness might cause errors, even in aircraft accidents. These processes include perceptual, memory, planning, assumption, and expectation processes, which can be summarized as internally oriented mental processes, goal-directed processes, and ongoing evaluation [3, 4].

The human brain is a large-scale interconnected organization [5]. Network approaches are useful for understanding how the brain function is organized. Many stable, intrinsic large-scale brain networks have been identified. Among them are three core neurocognitive networks: default mode network (DMN), central executive network (CEN), and salience network (SN) [6, 7]. These three networks play a critical role in human cognition.

We live in a world that is full of information. We must determine which information is salient and requires further processing. Task-based functional magnetic resonance imaging (fMRI) studies have found that the SN is the key brain network for salience detection [8]. The SN anchors the dorsal anterior cingulate cortex and bilateral insula cortex [9, 10]. It involves detecting behaviorally relevant stimuli and coordinating neural resources [9]. It also supports the representation and updating of the current salient information. The primary role of the SN is to switch between different brain connectivity states [11]. The core regions of the DMN are the medial prefrontal cortex, the precuneus/posterior cingulate cortex, and the bilateral parietal cortex [12]. Pilots have been found to exhibit increased functional connections within the DMN [13]. The DMN is always deactivated during stimulus-activation cognitive tasks and activated during the resting state [14]. The DMN appears to involve self-referential/internally oriented mental processes [15]. Meanwhile, the CEN involves the bilateral dorsolateral prefrontal cortex and the posterior parietal cortex [16]. It always shows strong activation during a variety of cognitively demanding tasks. The CEN is suggested to play an important role in goal-directed behavior [17, 18].

The SN and the CEN often coactivate together across a wide range of cognitive tasks, whereas the DMN always shows a decreased activation [14]. It has been proposed that the SN controls the interactions between external-task-directed (CEN) and self-related (DMN) processes based on salience decisions [19]. These three networks are supposed to be a triple-network model of the brain’s architecture [6]. Moreover, the variation in the engagement and disengagement of these three brain networks is crucial in many psychiatric and neurological disorders [6]. The relationships between the three networks provide the basis for understanding the brain mechanism.

The triple-network model could comprehensively reveal the brain mechanism; hence, the current study aimed to examine the functional connectivity properties of the three networks in pilots. We used resting-state fMRI in pilots and healthy controls who worked on the ground. The same was successfully used in drivers [20, 21]. A resting-state activity represents an intrinsic brain activity and can be used to detect the patterns of a coherent intrinsic brain activity. Independent component analysis (ICA) was conducted herein on the fMRI data to identify the SN, DMN, and CEN. Functional connectivity is typically assumed as a static network. In fact, the neural synchronization of the brain may have subtle differences across time. Sliding window dynamic functional connectivity analysis was also conducted herein to detect the dynamic properties of the neural synchronization of the three large-scale brain networks. We hypothesized that the dynamic properties of the large-scale brain networks in civil aviation pilots are different from those in healthy controls.

## Materials and methods

### Participants

In this work, we report on resting-state fMRI data collected from 26 pilots on active duty and 24 education, handedness, and gender-matched healthy controls. The participants were recruited via online advertisements and given compensation. The exclusion criteria for all subjects included neurological illness, traumatic brain injury, substance-related disorders, and any metal implant in the body. All participants were fully informed about the procedure, and we obtained written informed consent. Detailed information of the participants can be found in our previous study [13].

This experiment was approved by the Ethics Committee of the University of Electronic Science and Technology of China (Chengdu, China) (No. 2018–042002).

### Data acquisition

The data were collected using a 3-T MRI scanner (DISCOVERY MR 750, GE) at the University of Electronic Science and Technology of China.

A three-dimensional spoiled gradient echo pulse sequence was used to collect axial high-spatial-resolution structural images (TR = 5.972 ms, TE = 1.972 ms, flip angle = 9°, matrix = 256 × 256, slice thickness = 1 mm, FOV = 25.6 cm × 25.6 cm, number of slices = 154).

The resting-state functional images were collected using a gradient-echo echo-planar imaging sequence. During the 510-s scanning, the subjects were lying quietly with their eyes closed while remaining awake (TR = 2000 ms, TE = 30 ms, flip angle = 90°, matrix = 64 × 64, slice thickness = 4 mm with no gap, FOV = 24 cm × 24 cm, number of slices = 35/volume).

### Data preprocessing

The SPM12 (Statistical Parametric Mapping 12, http://www.fil.ion.ucl.ac.uk/spm/) toolbox was used to conduct image pre-processing. The structural images were segmented into white matter, gray matter, and cerebrospinal fluid.

The first five scans of the resting-state fMRI data were discarded. The remaining images were slice-time- and head-motion-corrected, realigned, and coregistered to the structural images and transformed into a standard MNI space (3 mm × 3 mm × 3 mm). Subsequently, an 8-mm full-width at half-maximum Gaussian kernel was used to smooth the images.

The participants with excessive head motion (2 mm displacement in any direction or 2° spin in any direction) were excluded. The group head motion differences were compared. In addition, the head motion of each subject was analyzed as follows:

$$headmotion=\frac{\sum_{i=2}^{M}\sqrt{{\left(x_{i}^{1}-x_{i-1}^{1}\right)}^{2}+{\left(y_{i}^{1}-y_{i-1}^{1}\right)}^{2}+{\left(z_{i}^{1}-z_{i-1}^{1}\right)}^{2}+{\left(x_{i}^{2}-x_{i-1}^{2}\right)}^{2}+{\left(y_{i}^{2}-y_{i-1}^{2}\right)}^{2}+{\left(z_{i}^{2}-z_{i-1}^{2}\right)}^{2}}}{M-1}$$

M represents the total time point number; *i* is the certain time point; x^1^, y^1^, and z^1^ represent the translation parameters at a certain time point; and x^2^, y^2^, and z^2^ are the rotations.

### Group independent component analysis

Spatial ICA was conducted using GIFT software (Version 1.4b, http://icatb.sourceforge.net/). The minimum description length criterion was used to determine the number of independent components of the datasets from the 50 participants. Ultimately, 35 independent components were determined. Principal component analysis was conducted on the concatenated functional data of all subjects. The Infomax algorithm was used to decompose the data by independent component estimation. This operation was repeated for 20 times in ICASSO to achieve reliable results. Subsequently, dual-regression was conducted to back-reconstruct individual subject components into a single-subject space. Finally, each map was scaled to a Z-score.

We inspected the aggregate spatial maps and average power spectra of the independent components [22] to identify the relevant networks. Three experts rated the components from zero (certain artifact) to one (definite brain network) based on the principles that the components should exhibit primary activations in gray matter, which reflect networks of interest [22, 23], and low spatial overlap with known vascular, ventricular, and susceptibility artifacts. In addition, the power spectra of the components should be dominated by low-frequency fluctuations. The ratio of integral spectral power below 0.1 Hz to power between 0.15. and 0.25 Hz was used. This ratio should be greater than 3.

### Dynamic functional connectivity

Several methods can be used to map the dynamic functional connectivity, and the sliding window correlation is the most classical among them [24]. First, a fixed-length time window is used to divide the original fMRI data into a continuous series of fragments. Next, the Pearson correlation is computed between the three networks within each window. In the current study, we conducted a sliding window dynamic functional connectivity analysis using the Dynamic FNC Toolbox (v1.0a) in GIFT. In line with a previous study [25], the resting state data were divided using sliding windows of 20 repetition times (40 s) size with step by one repetition time (2 s). An additional L1 norm constraint on the inverse covariance matrix to promote sparsity in the graphical LASSO framework with 100 repetitions was imposed. Thus, each subject had 230 functional connectivity matrices. All functional connectivity matrices were then normalized using Fisher’s Z transformation.

### Clustering analysis

A *k*-means clustering algorithm with square Euclidean distance, 500 iterations, and 150 replicates was used on the functional connectivity matrices for all subjects. The centroids of these clusters, which can be treated as “states,” present the average patterns during scanning. The optimal number of clusters was estimated using the elbow criterion. Finally, a *k* of four was obtained using this method in a search window of *k* from two to ten [26].

We then conducted two sample *t*-tests [27] to identify the differences between the groups in each state. The results were corrected for multiple comparisons using false discovery rate (FDR) (p < 0.05).

## Results

### Participants

One pilot was excluded because of excessive head motion. Subsequently, 25 pilots and 24 healthy controls were included in the final analysis. **Table 1** shows the demographic parameters for the remaining subjects.

**Table 1:** Demographic characteristics.

	Pilots (N = 25)		Controls (N = 24)		Significance	
	M	SD	M	SD	T value	p Value (two-tailed)
Age (years)	25.92	3.12	29.33	4.02	−3.17	0.003*
Sex (% male)	100%		100%			
Education (years)	16		16			
Right hand (%)	100		100			
Total flight hours	1233.44	2390.04				

M: mean value; SD: standard deviation

### Networks of interests

**Fig 1:**
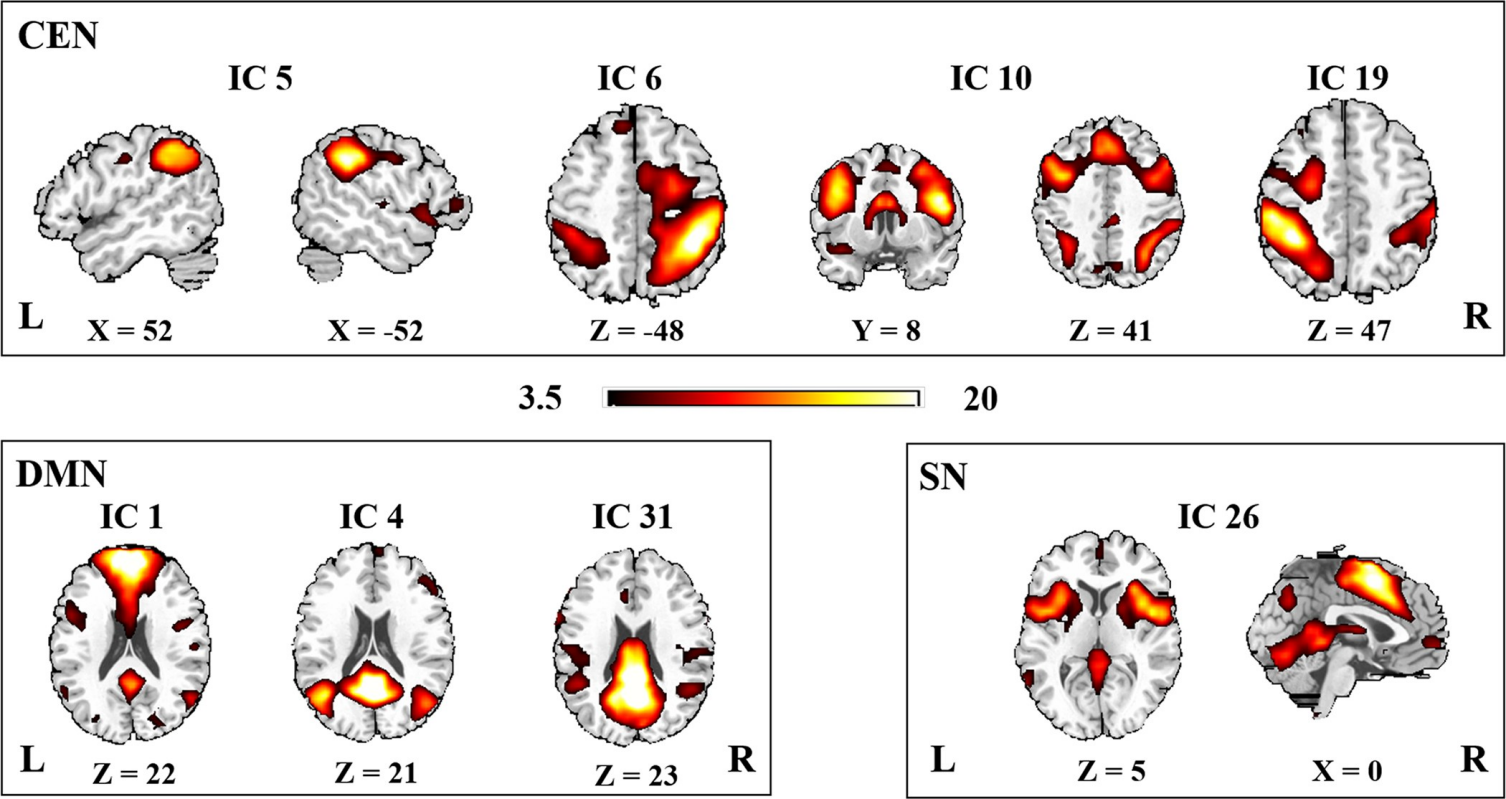
Spatial maps of the eight identified intrinsic connectivity networks (one-sample *t*-test, p < 0.001, uncorrected) sorted into three subcategories.

Intrinsic connectivity networks and their associated time courses were decomposed using the spatial ICA. From a total of 35 components, 8 were chosen as our networks of interest, among which, 3 components represented the DMN; one component represented the SN; and four represented the CEN according to our previous studies [7, 28, 29]. Fig 1 shows the spatial maps of these networks. **Table 2** presents the primary regions of each network.

**Table 2:** Brain regions with peak value of the networks.

Network		Center (MNI)			Peak T value	Brain regions (AAL)
		x	y	Z		
DMN	1	−9	54	21	29.03	Left superior frontal gyrus
	4	−3	−51	9	34.91	Left precuneus
	31	−3	−63	24	32.98	Left precuneus
SN	26	3	3	48	24.03	Anterior cingulate gyrus
CEN	5	57	−39	33	24.90	Right supramarginal gyrus
	5	−60	−36	36	20.39	Left supramarginal gyrus
	6	39	−45	45	25.59	Right inferior parietal lobe
	10	−51	12	21	27.76	Left inferior frontal gyrus
	19	−54	−30	39	26.08	Left inferior parietal lobe
	19	57	−30	42	10.71	Right inferior parietal lobe

### Differences between groups in the dFNC

**Fig 2:**
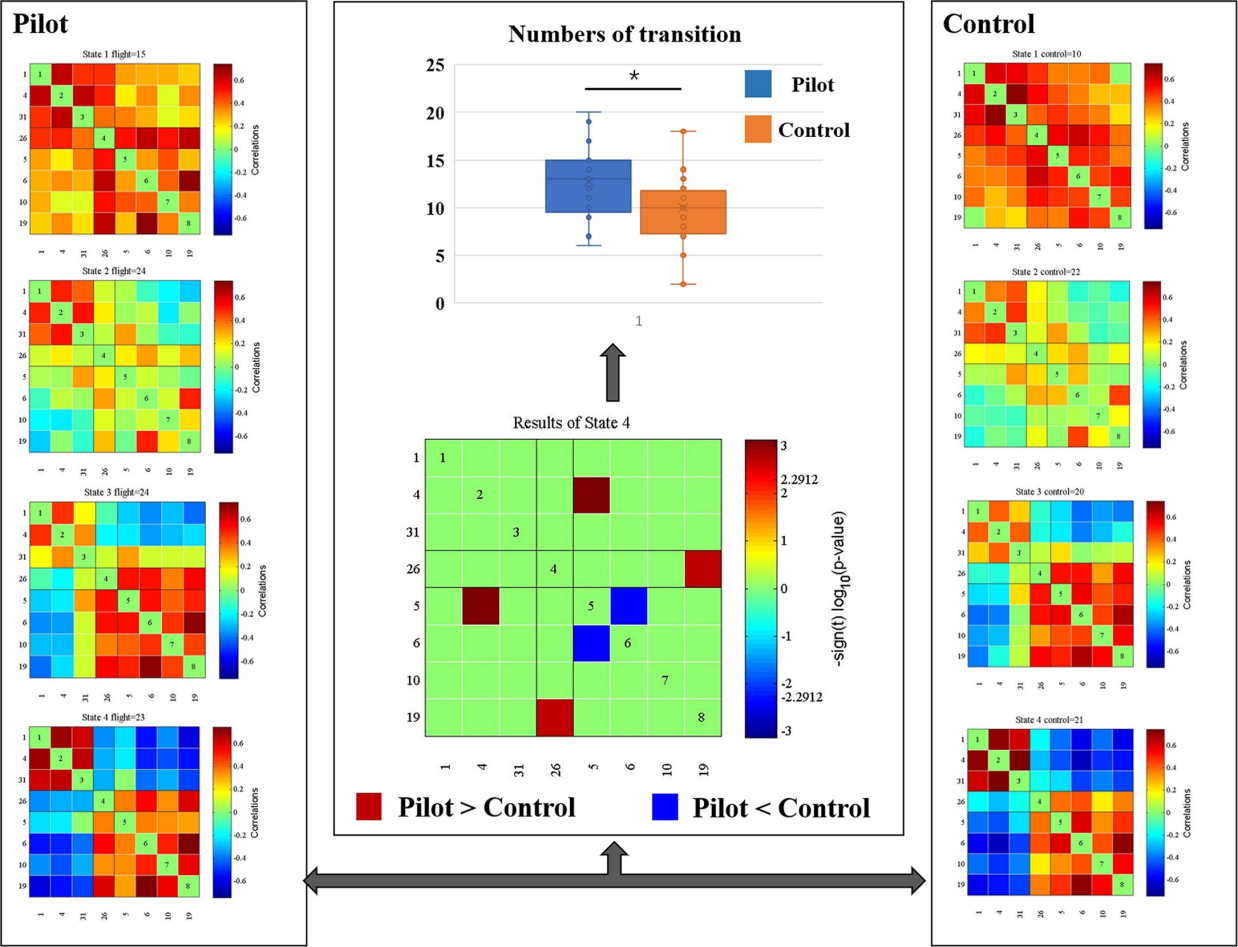
Differences in the dynamic functional network connectivity between groups. The left and right parts represent the median connectivity matrices (centroid) in each group. The middle part represents the group differences (FDR corrected, p < 0.05).

We identified four dynamic patterns of functional connectivity states. Fig 2 depicts the group-specific medians for each state. Significant group differences were observed only in State 4 (Fig 2). Two stronger between-network connections were found in the pilots compared to the healthy controls (i.e., CEN–DMN and CEN–SN). We also identified one decreased within-network connection in the pilots (i.e., CEN–CEN).

In addition, significant group differences were identified in the number of transitions after controlling for age. The pilots (12.72 ± 3.76) changed more frequently between brain states than the controls (10 ± 3.98) (p = 0.018).

## Discussion

By employing the dynamic functional connectivity analysis, the current study is the first to identify the differences in the dynamic connectivity between the pilots and the controls. Our results revealed that the pilots exhibited an increased interaction of the CEN with the DMN and SN along with a decreased interaction within the CEN. They also exhibited a higher number of transitions.

In the human brain, lower-order brain systems, such as the primary motor and sensory systems, always have limited connections to other brain systems. Higher-order systems, such as the CEN, SN, and DMN, which involve internally guided, higher-order mental functions, have significantly more functional connections with other brain modules [30]. Previous studies have found that the DMN always has high within-network connectivity and high between-network connectivity, while the SN and the CEN always have high between-network connectivity and relatively low within-network connectivity [31]. In addition, long-term cognitive training also led to an increased interaction among the three networks [7]. These functional connectivity profiles may be crucial for optimal cognitive functioning [32] and may be the optimal functional configuration of the human brain. Meanwhile, during development, the CEN becomes increasingly incohesive, which means it slowly reduces the within-network connectivity. This characteristic may enable the system to have different subnetworks to enable more diverse functions [33].

In the current study, the pilots exhibited a decreased within-network functional connectivity in the CEN and enhanced functional connections among the CEN, SN, and DMN. The incohesive CEN in the pilots might enable the network to have more diverse functions. Meanwhile, the CEN, SN, and DMN are increasingly associated with other brain systems during development [31]. An increased between-network connectivity may correlate with the general cognitive performance. The aviation environment is a dynamic and high-consequence environment, and flying is a complex cognitive task. Flying included diverse tasks, such as manipulation, navigation, communication, cross-checking, monitoring, strategy planning, and conflict resolution. Pilots must always perform several tasks in a limited time and, sometimes, simultaneously. They should acquire, integrate, and respond to various task-related cues to make accurate assessment and speedy judgment. They conduct continuous cognitive transitions during work. Thus, the different functional properties of pilots may be related to their increased functional dynamics.

In the current study, the pilots made more state transitions than the controls. A higher number of transitions may be associated with greater functional dynamics and cognitive flexibility [34]. The reduced transition probability between states was associated with the immature and inflexible dynamic interactions between these three networks [35]. Moreover, the reduction in the transition between states was associated with patients with demented Parkinson’s disease [36]. A higher number of transitions in the pilots in our results might suggest that they have better functional dynamics and cognitive flexibility.

The present study has a few shortcomings. First, the age difference between the groups may have some effect on functional connectivity. The age range of the participants herein was 23 to 36. The functional connectivity between the brain areas was relatively stable. We expected that the age difference would not have a major impact on the results. Second, relevant cognitive tests were lacking. The explanation of our results could only be based on speculation. Task-based fMRI studies are needed to provide complementary information. In addition, we aim to foster a long-term relationship with some airlines, and the findings of the current study should be further confirmed with a larger sample.

## Conclusions

In summary, this is the first study to assess the dynamic connectivity patterns in civil aviation pilots. Consequently, we demonstrated altered dynamic functional properties in pilots. The pilots exhibited increased between-network functional connectivity and decreased within-network functional connectivity in the CEN. In addition, the temporal properties of functional dynamics (number of transitions) increased in the pilots compared to the healthy controls. Previous studies have found a relationship between these differences and a better cognitive ability. These differences in the pilots might suggest that they have better functional dynamics and cognitive flexibility. Our findings imply that the dynamic functional connectivity analysis could be a useful imaging biomarker to monitor the changes in the brain functions of pilots.

## Supporting information

S1 Fig (TIF)
