# Supplementary figures and images for: Increased functional dynamics in civil aviation pilots: Evidence from a neuroimaging study
Source: PLoS One. 2020 Jun 18;15(6):e0234790. doi: 10.1371/journal.pone.0234790 (PMC7302522; doi:10.1371/journal.pone.0234790)

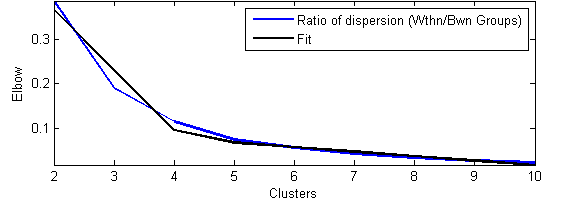

Supplement: S1 Fig — (TIF) [file pone.0234790.s001.tif]
